# Evaluating the Feasibility of Technology-Based Interventions in Disability and Rehabilitation: Definitions, Considerations, and Dimensions

**DOI:** 10.2196/79026

**Published:** 2026-04-14

**Authors:** Josh Shore, Amy S Hwang, Rosalie H Wang

**Affiliations:** 1 Rehabilitation Sciences Institute, Temerty Faculty of Medicine, University of Toronto, Toronto, ON, Canada; 2 Department of Occupational Science and Occupational Therapy, Temerty Faculty of Medicine, University of Toronto, 500 University Avenue, Toronto, ON, M5G 1V7, Canada, 1 4169468566; 3 Centre de recherche de l'Institut universitaire de gériatrie de Montréal (CRIUGM), CIUSSS Centre-Sud-de-l'île-de-Montréal, Montréal, QC, Canada

**Keywords:** rehabilitation, disabled persons, self-help devices, biomedical technology, feasibility studies, research design

## Abstract

Technology-based interventions in the field of disability and rehabilitation, which serve assistive, therapeutic, and/or service delivery functions, are considered complex due to the skills required of providers and recipients, degree of individual tailoring, and diversity of use settings. Feasibility studies are an important step in the evolution of complex interventions that can help refine the intervention, inform implementation, and prevent wasted resources. However, guidance is lacking regarding specific considerations for feasibility studies of technology-based interventions in disability and rehabilitation, which leaves researchers and developers reliant on resources from other fields that do not address important technology properties. To advance the field, context-specific definitions, considerations, and evaluation dimensions must be explicitly outlined to ensure that feasibility studies are constructively designed to meet the unique needs of these interventions. In this viewpoint article, we (1) propose a definition and framework for feasibility studies within the specific context of technology-based disability and rehabilitation interventions, (2) highlight important and unique imperatives for feasibility studies of these interventions, and (3) articulate relevant feasibility dimensions and associated evaluation criteria for these interventions. Building on previous work, we distinguish between feasibility studies, wherein we focus on iterative intervention refinement by addressing key development questions (eg, usability), and pilot studies, which are small-scale versions of a larger study that will evaluate intervention outcomes. Integrating previous typologies, we present 13 feasibility dimensions relevant to technology-based interventions and provide sample evaluation criteria, focusing on the intervention itself rather than study design considerations (eg, trial management). This information may be useful for research and development communities (academic, clinical, or industry) to inform comprehensive feasibility studies that examine unique aspects of technology-based interventions to promote real-world impact. This contribution encourages greater harmonization of terminology and evaluation methods to streamline interpretation and comparison across studies.

## Introduction

### Technology-Based Interventions in Disability and Rehabilitation

Innovative technology-based solutions are increasingly sought to meet the evolving needs of health and social service systems, particularly since the onset of the COVID-19 pandemic [1-3]. In the field of disability and rehabilitation, technology-based interventions involve structured use of technological devices or systems to achieve targeted goals related to health, function, or participation. Technology-based interventions can be broadly classified as serving assistive, therapeutic, and/or service delivery purposes [4]. Assistive products, such as wheelchairs, hearing aids, and communication devices, help optimize a person’s functioning to reduce disability [5]. Therapeutic interventions restore or improve function through the use of technologies including robotics, alternative reality systems, and exercise-based video games. Technology-mediated service delivery models replace or augment existing services to improve care access or maximize efficiency through approaches like telerehabilitation, web or mobile apps, and smart home monitoring systems. Technology-based interventions encompass the core technology products, such as the devices, software, or platform, as well as services (eg, training) and systems (eg, smart home system) that enable use of the intervention. Target users of such interventions may include rehabilitation patients, people with disabilities, clinicians, or care partners.

Regardless of intended function and target population, technology-based interventions are considered complex for several reasons. They often require providers and recipients to learn specific skills and necessitate customization or tailoring to individuals and/or specific contexts or settings of use [6]. Varying these factors may also engender technical complexity (eg, privacy, data security, reliability, usability, and scalability) that needs to be managed. Rapid testing may help uncover design requirements that are not identified during initial design activities and guide iterative intervention refinement.

### Feasibility and Pilot Studies

Feasibility is a vital facet in the Medical Research Council (MRC) framework for complex intervention development and evaluation [7]. Dictionaries define feasibility as the possibility, capability, likelihood, or reasonableness that something can be made, done, or achieved [8,9]. In the context of health research, feasibility studies refer to preliminary research before a main study to determine whether future work can and should be done, and to inform the design of such work [10]. According to the MRC framework, feasibility studies should address both the intervention itself (ie, content and delivery), as well as study design and process considerations for future evaluation (eg, recruitment, data collection, outcome measurement) [7]. Common aspects of feasibility studies include a focus on process rather than outcomes, exploration of key areas of uncertainty, and evaluation of multiple dimensions based on predetermined success or progression criteria [11,12]. Thorough feasibility studies prior to larger-scale evaluation contribute to better interventions, stronger evaluation designs, greater likelihood of successful implementation, and fewer wasted resources [7,11].

Historically, the terms feasibility and pilot have been used interchangeably and inconsistently in the literature, causing confusion about their scope and objectives [13,14]. In response to this lack of terminological clarity and growing interest in the field, Eldridge et al [10] developed an international consensus–based conceptual framework for feasibility and pilot studies that has been widely cited. According to their framework, feasibility studies ask whether something can be done, if it should be done, and *how* it should be done. Pilot studies are considered a subset of feasibility studies that ask similar questions but do so by conducting a small-scale version of a future study (or parts of it) [10]. It is now generally accepted that feasibility studies represent an overarching type of research concerning whether something will work, with pilot studies as a subset that specifically aim to test the operation of a future larger study [10,14,15].

Rooted in phased clinical trial practices, the Eldridge et al [10] framework focuses on studies conducted in preparation for a randomized controlled trial (RCT). However, RCTs are often inappropriate for technology-based interventions in disability and rehabilitation due to practical challenges (eg, need for prolonged intervention use across diverse settings, difficulty matching the speed of technology advances), scientific limitations (eg, lack of masking, and limited generalizability to real-world contexts), and ethical concerns of withholding interventions with observable benefits (see Wang et al [4] for detailed description of these issues). Given the challenges associated with reliance on phased clinical trial practices, new approaches have been proposed to generate evidence for technology-based interventions in disability and rehabilitation [4,16]. An alternate field-specific conceptualization of feasibility studies is thus warranted.

### Feasibility Evaluation of Technology-Based Interventions

To address the challenges above, Wang et al [4] proposed the Framework for Accelerated and Systematic Technology-Based Intervention Development and Evaluation Research (FASTER), a three-phased approach to evidence generation informed by the MRC guidance: (1) development, (2) progressive usability and feasibility evaluation, and (3) scaled evaluation and implementation [4]. The aim of the feasibility phase is to progressively refine the intervention prototype through small-scale iterative usability testing and assessment of other process concerns (eg, acceptability, adherence, and intervention safety) [4]. By uncovering insights on how to improve intervention features, the feasibility phase is essential to promote future uptake and sustained use [4]. Within the feasibility phase, FASTER promotes a variety of research designs and methodologies to gain a comprehensive understanding of the intervention, its users, and their contexts of use. Teams are encouraged to progressively address issues identified in previous work and tailor the feasibility evaluation to key research questions, uncertainties, and intervention maturity [4]. However, no guidance is offered on what aspects of feasibility may be relevant.

To our knowledge, no existing frameworks delineate aspects of feasibility relevant for technology-based interventions. While previous work has provided guidance on the design, key features, and areas of evaluation for feasibility studies across various health-related fields (eg, pharmaceuticals [17], public health [12], psychology [11], and rehabilitation [18,19]), these resources do not account for aspects of feasibility related to interactions between users, technology, and the environment (eg, usability and technical performance), or unique considerations for disability and rehabilitation populations (eg, duration and diversity of intervention use). Specification is important since feasibility studies are applied differently across fields. Technical feasibility studies within engineering or computer science disciplines generally assess whether a system can be developed to perform its intended functions within defined constraints and are therefore typically undertaken in highly controlled settings. Within business settings, feasibility studies assess the likelihood of success for a proposed commercial product or service considering factors like market demand and financial viability. A comprehensive harmonizing framework is needed to provide field-specific guidance for feasibility studies of technology-based interventions, insofar as their potential to perform as expected and deliver intended outcomes.

A harmonized framework could promote more efficient and theoretically driven intervention design, development, and evaluation. Currently, researchers and developers resort to borrowing and “translating” concepts from sources in other fields—a time-consuming undertaking that requires deep familiarization with new literature. This translation leads to differences in interpretation and application of concepts, making it difficult to compare and consolidate across studies. Without unified and cohesive resources, research in this area remains conceptually and empirically fragmented, delaying the pace and quality of innovation. To advance the field amidst rapid digitization of tools, services, and programs, a comprehensive framework is needed that outlines feasibility considerations, dimensions, and evaluation criteria pertinent to technology-based interventions. Such a framework will help researchers and developers achieve consistency and comparability, providing a foundation to develop best practices for studying the feasibility of technology-based interventions for disability and rehabilitation.

### Aims

This viewpoint article aims to (1) propose a definition and framework for feasibility studies within the context of technology-based disability and rehabilitation interventions, (2) highlight important and unique imperatives for feasibility studies of these interventions, and (3) articulate feasibility dimensions and evaluation criteria relevant to these interventions. We focus on the feasibility of the intervention itself rather than study design and process considerations (eg, trial management) for which suitable field-specific guidance is available [18-21]. It is hoped that this discussion encourages comprehensive feasibility studies that examine unique aspects of technology-based interventions to promote real-world research impact.

### Authorship Identity Statement

For transparency, we highlight key identities of the authorship team informing this viewpoint. The authors have varied disciplinary backgrounds (kinesiology, occupational therapy, cognitive sciences, rehabilitation science, biomedical engineering, and computer science) and degrees of clinical, research, and lived experiences related to rehabilitation and assistive technologies. Our experiences span diverse populations (eg, traumatic brain injury, respiratory conditions, stroke, and dementia), technologies (eg, telerehabilitation, smart homes, mobility devices, artificial intelligence, robotic rehabilitation, and assistive devices), and user groups (eg, care recipients, partners, providers, industry, and policymakers), across the lifespan (children to older adults). These experiences shape our assumptions about the iterative multifaceted nature of technology-based intervention research and inform our interpretation of the literature.

## Defining Feasibility Studies of Technology-Based Interventions

Building on conceptual definitions from Eldridge et al [10] and FASTER [4], we propose the following adapted definitions of feasibility and pilot studies for application to the context of technology-based intervention research in the field of disability and rehabilitation (Textbox 1).

Together, feasibility and pilot studies aim to gather insights about intervention features and their delivery [4,11,19]. This involves a focus on issues related to the intervention (eg, practicality, usability, and safety) and its implementation, as well as gaining a comprehensive understanding of user experience with the intervention across use contexts [4,19,22]. Once an intervention is sufficiently developed and data support its feasibility, pilot studies may be undertaken as part of the scaled evaluation and implementation phase [4,11]. Pilot studies have a greater focus on outcomes and may be more likely to consider additional scientific, logistical, or study process factors relevant to the design of future larger definitive studies, such as recruitment and retention rates, data completion and quality, and effect size estimates for efficacy measures [11,19]. At this stage, technology-based interventions should be evaluated with target users in real-world settings to enable realistic measurement of use and abandonment, effects on health or social outcomes, and economic considerations [4,7]. Pilot studies can be particularly valuable to inform such research and scale evaluation across time periods, user groups, care contexts, or geographic locations.

The next section highlights imperatives for evaluating the feasibility of technology-based interventions in disability and rehabilitation, followed by a summary of potential feasibility dimensions and associated evaluation criteria. To reiterate, we focus on the feasibility of the technology-based intervention itself, rather than study design and process considerations for future larger evaluation studies. Hence, factors related to study procedures (eg, recruitment, data collection, research infrastructure) are not included. Readers are directed to other works that discuss these aspects of study feasibility for intervention research, which are readily applicable to technology-based interventions [17-20]. We recognize there is overlap between intervention and study feasibility, and as such, information presented below may be useful for either purpose.

Textbox 1. **Proposed definitions for technology-based interventions in disability and rehabilitation.**

**Feasibility study:** preliminary investigation of a concept of, a part of, or a whole new technology-based intervention to assess its viability and inform its iterative refinement by addressing uncertainty about key development and design questions, such as how well an intervention prototype can be used (ie, usability), whether it should be used, and how it should be used.

**Pilot study:** specific type of feasibility study that is a small-scale version of a part of or whole, larger future study that evaluates outcomes, such as the use, effectiveness, or impacts of a new technology-based intervention. Pilot studies may be conducted in either controlled or naturalistic real-world contexts and require the intervention to be administered. Pilot studies involve target end-users, or participants who have some of the characteristics or experiences of the target end-users or knowledge of the experiences and contexts of target end-users. Decisions about the study context and participants depend on factors such as technology readiness or ethical concerns (eg, anticipated risk to target end users).

## Imperatives for Evaluating the Feasibility of Technology-Based Interventions

Developing a new technology-based intervention is an iterative nonlinear process that often involves cycles of prototyping, evaluation, and refinement [4]. Several rounds of feasibility testing and design modification may be warranted before an intervention is considered for larger-scale evaluation. While most interventions require some form of feasibility study, the inclusion of technology and unique needs of disability and rehabilitation populations introduces additional complexities that merit consideration. Based on our collective research and clinical experiences, literature review, and application of the FASTER framework [4], we synthesize 10 imperatives that feasibility studies for technology-based disability and rehabilitation interventions should address. These imperatives relate to target user populations; technology and intervention design; and regulatory, policy, and funding constraints (Textbox 2). While some are not entirely unique to technology-based interventions, the inclusion of technology complicates how these imperatives are addressed.

Textbox 2. **Imperatives for feasibility studies of technology-based interventions in disability and rehabilitation.**

**Target user population needs**

1. Accounting for diversity in user groups and target aims.
  - Technology-based interventions in disability and rehabilitation may have several different user groups and various target health or functional outcomes. [7] The profile of individuals receiving these interventions may also be complicated by heterogeneity, comorbidities or concurrent needs, and involvement in multiple interventions[23].
  - Triangulation of multiple research methods and/or data sources provides a more complete understanding of feasibility and maximizes knowledge gained about the intervention from smaller sample sizes [24].
2. Identifying meaningful user outcomes for health and social function.
  - Technology-based interventions for disability and rehabilitation populations serve functions beyond the medical model (curing symptoms or impairments), addressing issues related to health and social outcomes, such as management of chronic conditions, participation, and social inclusion.
  - While the focus of feasibility studies is not to evaluate intervention efficacy, exploring mechanisms of action and identifying the potential impacts and meaningful outcomes for users can help refine intervention features and inform future studies [4].
3. Creating conditions for meaningful feedback from different user groups.
  - Multiple user groups may interact with or be impacted by rehabilitation and assistive technology interventions, such as persons with a disability or clinical condition, clinicians, caregivers, and family members.
  - Given that different user groups may have distinct needs, values, capacities, and perspectives, creative testing approaches may be needed to simulate the experiences of various groups using the intervention in real-world conditions for them to provide meaningful contextualized feedback.
  - There may be stigma associated with the intervention that requires care to ensure that users feel comfortable testing it and providing feedback.

**Technology and intervention considerations**

1. Navigating technological complexities.
  - Technology-based interventions in disability and rehabilitation may be complex due to the number of technology components encompassed and their flexibility (ie, customizability) [7], skills required by service providers and recipients,[7] degree of individual tailoring required [7], and diversity of use settings [7]. These factors are complicated by variable levels of digital literacy and technical expertise among service providers and recipients who must learn and adapt to the technology.
  - Diverse study designs and methods from technical and clinical disciplines are thus needed to generate evidence for the feasibility and benefits of technology-based interventions [4].
2. Evaluating technology properties in real-world settings.
  - Evaluating technical performance (eg, system performance and reliability) and usability is critical to the development of new technology-based interventions [25]. These factors should be considered in early feasibility testing and require unique approaches to evaluation.
  - At the feasibility stage, intervention performance and usability should ideally be explored beyond a controlled lab environment [4,25]. Real-world testing considers how technology properties (eg, components and functions) may be affected by environmental conditions such as lighting, flooring, or background noise.
3. Monitoring adaptations needed to accommodate user needs.
  - Modifications to a rehabilitation or assistive intervention and its application may be needed to accommodate individual user needs or specific use settings. Adaptability to enable such modifications is a key feature of implementation success that should be addressed in feasibility testing.
  - Monitoring the frequency, extent, and impact of intervention modifications at the feasibility stage can provide important information regarding adaptability and help identify overly rigid features as well as opportunities for increased flexibility.
4. Incorporating relevant support services.
  - Support services are often required to facilitate technology-based interventions, including eligibility and fit assessment, device customization, user training, product servicing or maintenance, and technical support [4].
  - Incorporating aspects of these support services during feasibility testing can provide a more realistic evaluation of how the intervention may function in real-world environments.
5. Capturing appropriate duration and diversity of use.
  - Technology-based rehabilitation and assistive intervention use is often not discrete but rather may be used for prolonged or lifelong periods in various settings (eg, hospital or care center, home, workplace, community) that may each be associated with unique privacy, ethical, social, or security concerns.
  - Evaluating feasibility at appropriate intervals in diverse real-world contexts is important to identify changes in user experience that may occur during activities with differing demands, in unique environments, or due to changing individual circumstances over time (eg, decline in health status or new social environment).

**Regulatory, policy, and funding constraints**

1. Navigating and obtaining regulatory approvals.
  - Some rehabilitation and assistive technologies may be classified as medical devices requiring regulatory approval (eg, Health Canada, Federal Drug Administration, and European Medicines Agency).
  - Depending on jurisdictional requirements, specific outcomes or data may be needed to obtain relevant approvals, documentation of which may be initiated at the feasibility stage (eg, history of risk management and safety plans).
  - Development teams should be aware of applicable device classifications and associated requirements so they can be addressed in subsequent studies.
2. Examining variability in funding and reimbursement structures.
  - Funding sources (eg, publicly funded health care, third-party insurance, and self-pay) and reimbursement policies for technology-based interventions vary across jurisdictions, meaning there may be uncertainty about optimal approaches to providing access and establishing commercial feasibility.
  - Data related to the cost of the intervention and/or potential cost savings may support market research and commercial feasibility studies to establish a clear pathway for providing access.

## Evaluating the Feasibility of Technology-Based Interventions in Disability and Rehabilitation

Here we outline our approach to consolidate potential areas of focus for feasibility studies of technology-based interventions in disability and rehabilitation. Broadly, we “translated” existing concepts from other fields to better fit technology-based interventions by addressing important considerations in this field (see Imperatives for Evaluating the Feasibility of Technology-Based Interventions) and identified additional areas where needed. The first author identified sources by searching Google Scholar and PubMed using keywords including “technology,” “intervention,” and “feasibility” and reviewing forward and backward references. Commonalities across sources were clustered, synthesized, and organized into dimensions that were reviewed by other authors and refined through discussion. Based on our knowledge and experiences in the field, we developed guiding questions for each dimension by adapting existing descriptions from the literature where possible and selected sample evaluation criteria. Throughout this process, conflicting ideas were iteratively resolved through deliberation until consensus was reached.

Most feasibility aspects included here are not new, but rather a collection of those described in other fields [11,12,17-20], which we curated and adapted to this unique context. In particular, we drew upon guidance from Bowen et al [12] who proposed 8 areas of focus for feasibility studies in public health research that are relevant to technology-based interventions: (1) acceptability, (2) demand, (3) implementation, (4) practicality, (5) adaptation, (6) integration, (7) expansion, and (8) limited efficacy testing [12]. These areas were a foundation upon which we supplemented additional properties relevant to technology-based interventions.

### Dimensions Overview

Table 1 summarizes feasibility dimensions for technology-based interventions with associated guiding questions and sample evaluation criteria. We use the term “dimension” to describe aspects of feasibility, while evaluation criteria refer to different types of information to address the guiding questions. These criteria include relevant constructs or properties for qualitative inquiry, specific measured parameters and outcomes, and potential analysis tools or frameworks. Each dimension is discussed within the subsections below, including literature examples to illustrate specific points. These dimensions encompass clinical, technical, and commercial properties, but are not exhaustive or prescriptive. Rather, it is a starting point for determining pertinent issues and research questions on an individual basis, which may be addressed in a single study or through the course of multiple studies. As some dimensions are highly interrelated and evaluation criteria may be relevant across multiple dimensions, readers are encouraged to select dimensions and criteria based on the unique context and maturity of their intervention, providing appropriate justification.

**Table 1.** Proposed feasibility dimensions for technology-based interventions with associated guiding questions and sample evaluation criteria.

Dimension	Guiding question	Sample evaluation criteria
Technical performance^a^	To what extent does the performance of a technology-based intervention satisfy its requirements and meet its intended goals over a specified period of time in a given context?	Accuracy Responsivity and responsiveness Reliability Sensitivity Error resolution Maintenance and repair
Safety	To what extent is a technology-based intervention associated with risk of harm?	Frequency and severity of adverse events Perceived safety Risk management framework
Tolerability	To what extent can a technology-based intervention be used without experiencing unacceptable discomfort?	Device comfort and fit Serial symptom assessment Ecological momentary assessment Use sessions terminated due to discomfort
Acceptability^b^	To what extent is a technology-based intervention judged by users and other interest holders as suitable, appropriate, or satisfactory towards addressing its intended aim(s)?	Satisfaction Perceived appropriateness Interest or intention to use Recommendations to others
Demand^b^	To what extent is a technology-based intervention likely to be used by its intended users for its intended purpose?	Engagement Sustained use and abandonment Perceived value Interest or intention to use Accessibility Availability of alternatives
Usability^c^	To what extent does a technology-based intervention enable users to accomplish its stated goal(s) effectively, efficiently, and to their satisfaction?	Usability attributes Performance measures (eg, tasks completed, errors) Behaviors or affective responses (eg, observed or verbalized) Perceived usability Degree of exertion, demand, or workload
Adaptability^b^	To what extent can a technology-based intervention be modified to meet the needs of diverse users and use contexts?	Frequency, extent, and impact of device modifications Changes in user experience or impact resulting from modifications Protocol deviations Device fit assessments User settings and configuration Accessibility features
Practicality^b^	To what extent can a technology-based intervention be used in its intended setting(s) given resource constraints such as space, equipment, personnel, and time?	Space required for use and storage Duration and ease of equipment set-up and take-down Compatibility with existing equipment Personnel and training requirements
Implementation^b^	To what extent can a technology-based intervention be deployed and used as intended in environments with varying levels of control?	Adherence Fidelity Contexts of use Implementation barriers and facilitators
Integration^b^	To what extent can a technology-based intervention be incorporated into existing systems?	Perceived fit within organizational capacities and workflow Fit within organizational culture Perceived fit within daily routine Sustainability
Impact^b^	To what extent does a technology-based intervention show promise in eliciting positive changes for its target population?	Changes in clinical measures Appropriateness of measures Maintenance of changes Perceived benefits Caregiver burden System efficiency
Ethicality	To what extent is a technology-based intervention aligned with ethical values?	Bioethics (eg, Principlism) Technology ethics Prognosis Research Strategy framework Model for the Ethical Evaluation of Socio-Technical Arrangements framework Reflective questioning Socratic approach
Commercial viability	To what extent does a technology-based intervention show promise towards being economically viable for commercialization?	Production and operation costs Cost-effectiveness Economic modeling Product-market fit

a Adapted from Garrett et al [26]

b Adapted from Bowen et al [12]

c Based on ISO 9241-11:2018 usability definition

### Technical Performance

Users of technology-based interventions rely on systems that perform consistently to achieve their goals. Technical flaws and system errors are frequently cited as barriers to technology adoption [27,28]. Technical performance examines how well a technology-based intervention satisfies its functional requirements and meets its intended goals over a specified time period in a given context. This reflects aspects of technology performance evaluation from systems and design engineering [29]. Whereas usability focuses on interaction between the user and the technology, technical performance is more concerned with the function of the technology itself.

In systems engineering, technical performance measures refer to measurable qualities that determine whether a system satisfies key technical requirements [26]. Relevant technical performance measures for a project depend on the type of technology and its intended functions. Table 2 summarizes select technical performance measures with examples from interventions in disability and rehabilitation. While these properties may be initially addressed during intervention development, the feasibility stage extends performance evaluation to real-world contexts to identify features requiring refinement [4].

**Table 2.** Select technical performance measures relevant to technology-based interventions in disability and rehabilitation.

Measure	Definition^a^	Example
Accuracy	The closeness of agreement between an observed value and an accepted reference value (ground truth).	In a feasibility study of a sensor motion detection system for fall prevention in hospitalized older adults, Ferrari et al [30] assessed the accuracy of sensor output data by establishing its correlation with observer ratings of participant movement from video recordings.
Responsivity and Responsiveness	How well a system adjusts to commands or cues promptly.	Fang et al [31] evaluated the responsiveness of a Rotational Orthosis for Walking with Arm Swing system by assessing whether a measurable movement was produced that was close to the user’s target joint trajectory.
Reliability	Likelihood of a system performing its intended function without error for a given period of time under a specified set of conditions.	Awad et al [32] assessed the reliability of the ReWalk ReStore soft robotic exosuit by measuring device malfunctions during usage.
Availability	Likelihood that a system is operational at a given point in time under a given set of environmental conditions.	Marschollek et al [33] assessed the technical feasibility of a smart home monitoring system by measuring the downtime of installed systems in relation to their up-times.

a According to American Society for Quality [34].

### Safety

Several authors identify safety as a critical component of intervention feasibility, and it is among the most common measures reported by feasibility studies in rehabilitation research [20]. Safety refers to the risk of harm imposed on intervention recipients or others affected by the intervention [35]. In some cases, safety is closely related to technical performance. “Design for safety” is an approach whereby products and systems are purposefully engineered to prioritize the safety of people who interact with them, with specific features designed to proactively address safety risks [36]. For example, a robotic exoskeleton could be made “fail-safe” by defaulting into a safe system state upon detecting an operational error to prevent injury caused by excessive force applied to a limb [37]. Thus, intervention safety may depend on appropriate technical performance.

Safety is typically operationalized as the extent to which an intervention is associated with adverse events, such as injuries or disease exacerbations. Measuring the frequency and severity of adverse events provides information about the risks and potential harm of an intervention in a given setting. Defining adverse events prior to commencing evaluation helps ensure relevant incidents are captured and properly managed. For technology-based interventions, researchers may wish to evaluate users’ ability to complete tasks safely while using the device. This can be accomplished through behavioral observation and coding safety incidents and near misses. Perceived safety from the perspective of users and members of their support system may also provide valuable insight, particularly for devices that involve loss of control (eg, robotic exoskeletons), or those that target vulnerable populations like people with dementia.

Wang et al [38] provide an example of multidimensional safety evaluation in a study evaluating contact sensors for powered wheelchairs among older adults with dementia [38]. A multiple single-subject mixed methods design was used, involving quantification of safety-related incidents observed by the investigator during testing with older adults in nursing homes, and qualitative exploration of perceived safety via debrief interviews with participants and focus groups with staff. Investigator observations showed that while the sensors successfully detected most obstacles, minor design faults (eg, gaps between panels) led to some performance errors, and more sensors were needed for complete environmental coverage to minimize injury risk. The qualitative analysis revealed that resident and staff perceptions of safety were mixed, leading the authors to identify a need for further safety improvements by increasing sensor reliability and coverage [38]. This study demonstrates the close relationship between safety and technical performance and underscores the importance of assessing and redesigning for safety when developing technology-based interventions for people with cognitive impairment.

### Tolerability

Related to safety is the concept of tolerability. The US Food and Drug Administration defines tolerability as the degree to which adverse effects can be tolerated by participants [35]. For technology-based interventions, we conceptualize tolerability as the degree to which users can engage with the intervention without experiencing unacceptable discomfort. Tolerability is particularly important for interventions that are associated with discomfort, such as neuromodulation [39], brain-computer interfaces [40], and prosthetic devices [41], although it may also be relevant for other types of interventions that serve populations with sensitivities (eg, chronic pain, headaches, and autism spectrum disorder). It has important implications for other dimensions such as acceptability, usability, demand, implementation, and ultimately adoption. For example, device fit and comfort are critical factors in long-term use and abandonment of upper limb prosthetics [41]. Assessing tolerability can also help determine appropriate treatment dosage.

Self-report measures are useful to capture side effects or changes in participants’ condition while interacting with the intervention. For example, symptoms or discomfort can be rated before, during, and after intervention use with condition-specific instruments, or generic subjective rating scales (eg, Visual Analog Scale, Visual Rating Scale, Numeric Rating Scale, and Borg Scale) [42]. Validated device-specific comfort questionnaires are also available for some interventions, such as wheelchairs [43] and wearable computers [44]. When testing interventions in real-world settings, ecological momentary assessment (EMA) can be a useful approach. EMA involves repeatedly sampling participants’ current condition in real-time within their natural environment [45]. Technology-based interventions offer unique opportunities to embed EMA (eg, mobile app prompts), allowing teams to gather detailed and context-rich data on participant discomfort, stress, fatigue, and frustration. By assessing users’ condition at various time points in everyday environments, EMA can provide valuable insight into factors that influence tolerability and help monitor cumulative stress from long-term use.

Some objective methods of assessing comfort have been described. Physiological measures (eg, heart rate, skin conductance, and muscle activity) can identify adverse stress responses associated with the intervention. Pressure sensors and movement analysis techniques have been used to capture compensatory movements that indicate discomfort [42]. Finally, observational analysis of participants’ behavior during the intervention can help identify signs of discomfort, fatigue, or frustration [42].

Intervention usage (eg, frequency, duration, or maintenance) is sometimes reported as a measure of tolerability by indicating whether demands are appropriate, or if participants avoid the intervention due to discomfort. In such cases, it should be clear why usage metrics are framed as measures of tolerability rather than engagement or adherence, and how information about tolerability is inferred. For example, a study on a smart glasses social communication aid for people with autism assessed tolerability based on participants’ ability to wear the device during coaching sessions [46]. The authors were interested in how well participants tolerated the smart glasses due to their sensory and cognitive challenges, rather than whether they chose to use the smart glasses (ie, engagement) or comply with a prescribed treatment plan (adherence).

A good example of tolerability evaluation can be found in a case study from Ryan et al [47] on the integration of transcranial direct current stimulation (tDCS) into a pediatric inpatient brain injury rehabilitation program [47]. Individual session tolerance was monitored using pre- and postratings of common tDCS side effects (eg, headache, fatigue, itching, and tingling). A postintervention Tolerability Questionnaire [48] was also used to assess tolerance across the study period, which involved the participant ordering a list of common activities from most to least enjoyable (eg, playing games, watching TV, going to the dentist, and getting a needle), and rating their tDCS experience relative to those activities. With minimal symptom exacerbation within sessions and a favorable rating of tDCS relative to other activities, the authors concluded that tDCS was tolerated well by the participant and could be incorporated into inpatient rehabilitation [47].

### Acceptability

Acceptability is a foundational aspect of feasibility that describes how individuals involved in an intervention react to it [12]. While empathizing with users to understand their needs and preferences is a vital step in designing an intervention, perspectives may change once users actually experience it, underscoring the need for user involvement throughout development and evaluation [4]. According to Nielsen [49], technology is accepted when it satisfies the needs and requirements of its users and other interest holders. Thus, acceptability can be defined as the extent to which users of a technology-based intervention and other interest holders judge it to be suitable, appropriate, or satisfactory for addressing its intended aim. Acceptability is an important predictor of use behavior that precedes technology adoption [25]. Ultimately, successful implementation relies on procedures that are acceptable and meet the needs of individuals who deliver, receive, or otherwise engage with an intervention [11,50].

Efforts have been made to better understand and operationalize acceptability. Several theories have been proposed to explain technology acceptance, the most prominent of which include Technology Acceptance Model (TAM and TAM2), Combined Technology Acceptance and Theory of Planned Behavior Model, Motivational Model, and Innovation Diffusion Theory [51]. Combining principles from many models, the Unified Theory of Acceptance and Use of Technology (UTAUT and UTAUT2) posits that acceptance and intention to use technology are dependent on one’s beliefs about performance expectancy (utility toward one’s goals), effort expectancy (ease of use), and facilitating conditions (organizational and technical support), as well as subjective norms regarding their use of the technology, all of which are moderated by gender, age, experience, and voluntariness of use [52]. For health care interventions, Sekhon et al [50] developed the Theoretical Framework of Acceptability, which conceptualizes acceptability through 7 constructs: (1) affective attitude, (2) burden, (3) perceived effectiveness, (4) ethicality, (5) intervention coherence, (6) opportunity costs, and (7) self-efficacy [50]. It is clear that the acceptability of technology-based interventions in health care is complex and multidimensional, with several interacting factors related to beliefs and attitudes that overlap with other feasibility dimensions.

Satisfaction is considered an indicator of acceptability that can be measured quantitatively using questionnaires [50]. Sekhon et al [50] developed the Theoretical Framework of Acceptability Questionnaire, a general measure reflecting the Theoretical Framework of Acceptability constructs that can be adapted for various health care interventions [53]. Other generic satisfaction scales include the Client Satisfaction Questionnaire [54], Acceptability of Intervention Measure [55], and Intervention Appropriateness Measure [55]. For technology-based interventions, the TAM questionnaire evaluates acceptance based on perceived usefulness and ease of use [56]. Textbox 3 highlights acceptability and satisfaction questionnaires relevant to technology-based interventions [4]. Comprehensive reviews of satisfaction and acceptability tools for assistive technologies [57] and mobile health programs [58] are also available.

Textbox 3. **Sample acceptability and satisfaction questionnaires relevant to technology-based interventions in disability and rehabilitation.**

**Generic health care intervention measures**

- Theoretical Framework of Acceptability Questionnaire [53]
- Client Satisfaction Questionnaire [54]
- Acceptability of Intervention Measure [55]
- Intervention Appropriateness Measure [55]

**Generic technology measures**

- Technology Acceptance Model Questionnaire [56]
- Unified Theory of Acceptance and Use of Technology Questionnaire [59]

**Assistive technologies**

- Quebec User Evaluation of Satisfaction with Assistive Technology 2.0 [60]
- Almere Technology Acceptance Questionnaire [61]
- Remote Monitoring Satisfaction Survey [62]
- Satisfaction with Prosthesis Questionnaire [63]
- Satisfaction with Amplification in Daily Life Questionnaire [64]

**Therapeutic technologies**

- Physiotherapy Mobile Acceptance Questionnaire [65]
- Exergame Enjoyment Questionnaire [66]
- Virtual Rehabilitation User Satisfaction Evaluation Questionnaire [67]
- Immersive Experience Questionnaire [68]
- Questionnaire for User Interaction Satisfaction [69]

**Telehealth and mobile service delivery**

- Telemedicine Satisfaction Questionnaire [70]
- Telemedicine Satisfaction and Usefulness Questionnaire [71]
- Telehealth Satisfaction Scale [1-3]
- Service User Technology Acceptability Questionnaire [72]
- Website Satisfaction Scale [73]
- Digital Health Acceptability Questionnaire [74]

Although satisfaction questionnaires are simple quantifiable measures, qualitative methods often elicit a deeper understanding of user experiences and acceptability at the feasibility stage [11,75]. Interviews and focus groups can be conducted after users have tested an intervention to gather perspectives regarding appropriateness of the intervention and delivery approach to inform iterative refinement. Qualitative inquiry is particularly valuable to probe the unique and nuanced aspects of technology acceptability in the field of disability and rehabilitation, such as social implications of observable assistive technologies across use contexts.

Importantly, intervention maturity and stage of development influence acceptability. For instance, early stages may involve low-fidelity intervention prototypes that users trial on a single occasion and must imagine the experience of living with in real-life circumstances. Conversely, high-fidelity prototypes in later stages allow users to trial the intervention in real-world environments, over longer periods, and in more diverse contexts. Acceptability should thus be viewed as a dynamic attribute that warrants consideration throughout evaluation.

### Demand

Technology innovations in disability and rehabilitation are designed to address unmet needs by providing a new solution or service not available through conventional means. However, the likelihood that an intervention will be used to serve this function cannot be fully understood until in the hands of its target population. Based on interaction and experience with a prototype, demand evaluates the likelihood that a technology-based intervention will be used by intended users [12]. Demand is closely tied to acceptability, as users are more likely to engage with an intervention that is deemed suitable or satisfactory. By estimating future use, demand also has implications for the commercial viability of technology-based interventions.

A relatively straightforward indicator of demand is user engagement. Cavanaugh [76] describes engagement as efforts by users to start and continue with an intervention. Engagement differs from adherence in that it implies an active and volitional choice to interact with an intervention in a meaningful way, rather than passive compliance with a prescribed plan. Therefore, it reflects the extent to which a technology-based intervention is likely to be used, particularly for those that involve free use in uncontrolled contexts, such as home-based exergames, virtual reality programs, and assistive devices. Evidence across various types of technology-based interventions shows that user engagement is an important predictor of future use and intervention success [77]. In digital health research, engagement has been conceptualized as a multidimensional construct involving observable behavior (ie, usage parameters) and subjective experience characterized by attention, interest, and affect [78]. Behavioral usage parameters relevant to gauging demand include the frequency, duration, and depth (ie, variety or intensity) of usage [78]. For interventions that use websites, mobile apps, gaming systems, or other digital devices, these metrics may be captured automatically through system logs. The User Engagement Scale measures engagement experience based on aesthetic appeal, focused attention, novelty, perceived usability, felt involvement, and endurability [79].

Engagement should ideally be monitored longitudinally, as diminished use and abandonment are common in digital health and assistive technology interventions [80,81]. While engagement may be high initially, it often tapers over time as novelty fades. Considering this “law of attrition,” Eysenbach [81] recommends analyzing determinants of engagement with eHealth programs and characteristics of subgroups that maintain and discontinue engagement [81]. Feedback from participants regarding perceived value of the intervention and interest in continuing its use in their routine practice or daily activities can help understand demand. Qualitative inquiry is valuable to obtain a nuanced understanding of engagement by addressing questions such as *how*, *why*, and *when* users choose to engage with the intervention, and to appreciate psychological or social experiences of interacting with the intervention. This can help drive intervention refinement to promote more meaningful and sustained use, ultimately increasing demand.

### Usability

Rehabilitation and assistive technology interventions aim to provide a useful and *usable* tool to accomplish the goal of optimizing function and reducing disability. According to the International Organization for Standardization (ISO) 9241-11:2018, usability refers to how well a system, product, or service can be used by its target users in a specific context to achieve their goals with effectiveness, efficiency, and satisfaction [82]. Usability is a common area of evaluation for rehabilitation and assistive technologies with important implications for their acceptance, adoption, and effectiveness [83,84]. It is a key component of feasibility studies that is particularly important for iteratively refining intervention features [4].

The ISO emphasizes the multidimensional nature of usability, highlighting effectiveness, efficiency, and satisfaction as key elements [82]. Similarly, Nielsen [85] identifies 5 usability heuristics in his textbook Usability Engineering: (1) learnability, (2) efficiency, (3) memorability, (4) errors, and (5) satisfaction. Building on these conceptualizations, Quesenbery [86] coined the 5E’s of usability for user experience design: effectiveness, efficiency, engagement, error tolerance, and ease of learning. Table 3 summarizes these usability attributes. While not exhaustive, these attributes are a useful starting point for evaluating usability.

**Table 3.** Usability attributes and definitions.

	Usability attributes	Definitions
ISO Standard 9241-11:2018 [82]	Effectiveness	Accuracy and completeness with which users achieve intended goals or outcomes
	Efficiency	Resources used (eg, time, human effort, cost, and materials) relative to results achieved
	Satisfaction	Extent to which the user’s physical, cognitive, and emotional responses that result from the use of a system meet the user’s needs and expectations
Nielsen’s Usability Engineering [85]	Learnability	How easily a technology can be learned such that the user can rapidly begin to use the technology to perform the necessary tasks
	Efficiency	How well the user can achieve a high level of productivity with minimum resource requirements (eg, time, money, effort)
	Memorability	How easily the user can remember how to use the technology such that they can return to the product easily with minimal relearning after a period of disuse
	Errors	The extent to which errors are minimized and catastrophic errors are avoided
	Satisfaction	How pleased the user is with their experience of the technology
Quesenbery’s 5E’s of Usability [86]	Efficiency	The speed with which the user can complete work accurately using the technology
	Engaging	How pleasant, satisfying, or interesting it is for the user to interact with technology
	Error tolerance	How well the technology prevents errors and helps the user recover from errors that occur
	Easy to learn	How well the product supports the user in initial orientation and deeper learning

Design engineering practices provide various approaches for evaluating usability. According to Nielsen [85], usability can be tested directly through behavioral measures (what people do) or indirectly assessed based on attitudinal beliefs of the user (what people think). Direct methods of usability testing consider how technology is actually used by measuring behavior or performance [25,85]. This can involve quantifiable performance metrics or qualitative methods such as participant observation and/or review of materials produced by the user [25]. Relevant performance measures depend on goals of the intervention and may include accuracy, task completion time, and errors (recoverable and unrecoverable) [85]. Think-aloud protocols, which involve users verbalizing their thoughts while interacting with technology, are a valuable strategy to identify usability problems with small samples. Finally, observational analysis techniques involve coding participant behavior while using the technology [85].

Indirect or attitudinal methods of usability evaluation explore user perspectives regarding their experience interacting with technology, which can be done through questionnaires, interviews, focus groups, or other methods [25,85]. Some argue these measures of user attitudes reflect technology acceptability rather than usability per se [25]. Nevertheless, subjective experience questionnaires are the most common method of usability evaluation across assistive and rehabilitation technology intervention studies [87,88]. Textbox 4 lists usability questionnaires for technology-based interventions. Qualitative feedback through posttesting interviews or focus groups is recommended to gain a deeper understanding of why usability problems exist and how they should be addressed [25,89].

The most appropriate usability evaluation methods depend on project context and intervention maturity. Available options also depend on the context in which the intervention is studied, for example, under natural settings in real-world target environments, scripted use within a controlled laboratory environment, or only perceptions and expectations about hypothetical use [89]. Combining direct and indirect measurement often provides a more comprehensive understanding of usability by not only identifying apparent usability issues but also exploring why they exist and how they can be resolved [25]. Early usability studies might benefit most from think-aloud, observation, and focus group methods within scripted use contexts to gather rich data targeting specific issues with small sample sizes [25]. At the feasibility stage, real-world use is important, and usability evaluation might involve a combination of performance measures, observation, questionnaires, and interviews [4]. For larger and longer-term outcome studies, performance measures are likely most informative [25]. Readers are directed to Liu et al [25] for a thorough discussion of usability measurement, including examples from rehabilitation and assistive technology interventions.

Textbox 4. **Example usability questionnaires for technology-based interventions.**

- System Usability Scale [90]
- Usefulness, Satisfaction, and Ease of Use Questionnaire [91]
- NASA Task Load Index [92]
- Telehealth Usability Questionnaire [92]
- mHealth Application Usability Questionnaire [93]
- Usability Scale for Assistive Technology-Wheeled Mobility [94]
- Usability Scale for Assistive Technology-Computer Access [95]
- Accessible Usability Scale [96]

### Adaptability

Technology-based interventions for disability and rehabilitation are characterized by diversity in user populations and use settings [4]. Device modifications or personalized intervention features may therefore be needed to accommodate individual users. Adaptability is the degree to which a technology-based intervention can be modified to meet the needs of diverse users and use contexts [12]. Evaluating adaptability can help define the scope of the target population, identify overly rigid features, and determine opportunities for increased flexibility to enable personalization.

Adaptability encompasses the frequency, extent, and reasons for intervention modifications, as well as any unique user needs that could not be accommodated through modifications. Adaptable interventions are expected to achieve similar outcomes despite changes in features or delivery format to accommodate individual needs [12]. Thus, it is important to assess the impact of modifications, including whether implementation processes, user experiences, and outcomes are consistent across modified versions [11]. Addressing adaptability also provides an opportunity to test the processes that enable personalized modifications, such as fit assessments, trial periods, technical support, and professional follow-up.

The importance of considering the adaptability of early prototypes is evident in a study from Pellichero et al [97], which evaluated the usability and usefulness of a blind spot sensor system for powered wheelchairs [97]. Although customization of sensor mounting, feedback configuration (ie, sounds, lights, or vibration), and sensor parameters (detection distances) were available through an associated smartphone app, the study was conducted with a standardized set-up preventing customization. The authors subsequently postulated that insufficient customization may have limited participants’ use of the system during the 2-month home trial period and influenced perceived usability and usefulness. Customization of the sensor system according to individuals’ abilities, preferences, and environment was thus recommended for future research and implementation [97]. This example illustrates the interrelatedness of feasibility dimensions, highlighting how adaptability has important implications for evaluating other aspects of intervention feasibility (eg, usability and demand). It also demonstrates how allowing opportunities for customization and assessing subsequent modifications during early-stage evaluations can help optimize interventions to accommodate diverse user needs.

### Practicality

The best of interventions will not be successful unless they are practical within user and organizational capacities. Practicality refers to how well an intervention can be used in its intended setting given resource constraints like space, equipment, personnel, and time [11,12]. Literature across an array of technology-based interventions consistently highlights practical factors as important barriers to clinical implementation [98-100]. Disability and rehabilitation interventions may be deployed in hospitals or care centers, clinics, homes, or other community settings that have variable resource capacities, making practicality an important consideration.

Physical factors related to practicality include space and equipment needs for intervention use and storage [98,100]. System interoperability and compatibility with existing equipment and infrastructure is also important. For example, whether an exergame deployed for home use is suitable for conventional recreational gaming systems and whether it successfully integrates with commercial wearable heart rate monitors. Personnel required to facilitate intervention use should be assessed, including the level of training and expertise needed [98,101]. Time limitations are a well-documented barrier to implementation of rehabilitation technologies, which can encompass the duration of training, set-up, use, and take-down [98,101].

### Implementation

To understand whether an intervention will work, it is important to explore the process of how it is delivered and received [102]. The implementation dimension evaluates how well a technology-based intervention can be deployed and used as intended in target settings with varying levels of control. This is important given the diversity of contexts in which technology-based disability and rehabilitation interventions are used [4]. Examining implementation helps understand how well the intervention can be delivered and what supports are needed to enable successful delivery [11]. It can also inform the training and support required for users to learn and apply the technology appropriately. The context of use, within a controlled lab environment, home, or community setting, is an important consideration to determine the conditions under which the intervention is likely to succeed and identify features requiring adaptability [4,11,102].

Process evaluations assessing implementation describe the quality or integrity with which an intervention is delivered by monitoring adherence and fidelity [102]. Adherence is traditionally defined as the extent to which the behavior of an intervention recipient corresponds with the prescribed program [103]. Whereas we describe *engagement* above as an active choice to interact with the intervention in a meaningful way, we use the term *adherence* in reference to whether participants use the intervention as prescribed in its delivery [76]. Adherence is a common measure among feasibility studies of rehabilitation interventions that can be quantified via self-report or observation as the proportion of intervention components (eg, appointments, training sessions, modules, and tasks) completed by participants relative to those prescribed [20]. These measures, which translate well to technology-based interventions serving therapeutic or service delivery functions and may be captured through automated system logs, can help infer the burden of intervention demands and appropriateness of procedures [19]. For assistive technologies, adherence is poorly defined [104] and infrequently reported [105]. In reporting guidelines for assistive technology outcomes, Tuazon and Jutai [105] identify frequency, duration, and continuance (or discontinuance) of use as measures of adherence. We argue that these usage metrics reflect adherence when the intervention is used *as* recommended, but may be more appropriately viewed as measures of engagement in conditions of free voluntary use. Given the apparent similarity and overlap between adherence and engagement, we encourage authors to thoughtfully consider and rationalize their application.

Fidelity refers to whether the intervention is delivered as intended [102]. Self-report checklists and/or observational methods may be used to assess the degree to which intervention delivery and use align with key features of the intended design. This includes components or ingredients of the intervention and the level of competence demonstrated by intervention providers [11], which can help determine the training required to achieve proficiency and fidelity. A benefit of technology-based interventions is the possible integration of screen, audio, or video recordings that capture user behavior, enabling assessment of fidelity or adherence. For interventions that require complex technology operation, user perceptions regarding the ease of achieving fidelity may be informative and can be assessed using the Agency for Healthcare Research and Quality Feasibility Assessment for Wide-Scale Implementation (Criterion 2) [106]. These approaches to fidelity assessment work well for technology-based interventions with therapeutic or service delivery functions, for which treatment theory can be readily defined and interventions can be manualized, allowing active ingredients to be monitored [107]. In a provocative discussion, Lenker et al [108] highlight historical inadequacy of fidelity reporting in assistive technology research, which they attribute to poorly defined treatment protocols, temporal delays between device use and evaluation, and lack of available measurement tools to capture intervention attributes. To strengthen the quality of assistive technology research, they recommend greater inclusion of treatment theory, better specificity in intervention characterization, and routine fidelity monitoring [108].

The distinction between intervention and study feasibility is particularly blurred within the implementation dimension. For example, the fidelity by which clinicians deliver a tele-rehabilitation intervention and the extent to which recipients adhere to the program will likely have implications for both the success of the intervention itself (ie, whether benefits are achieved by recipients), as well as the scientific integrity and outcome of research studies evaluating the intervention. A clear rationale for evaluating implementation should be specified based on development stage (eg, refining intervention or study procedures).

### Integration

Organizational support and alignment with system-level priorities are frequently cited as facilitators to technology implementation in disability and rehabilitation practices [98-101]. The integration dimension considers the “fit” of a technology-based intervention in terms of how well it can be integrated into existing systems within its target settings [12]. A focus on system-level integration is important as it likely affects implementation quality and eventual technology adoption. Importantly, there may be multiple systems into which a technology-based intervention must integrate. For example, therapeutic exergames may be initially introduced by therapists within a clinical setting and subsequently integrated into individual or family routines at home for continued practice. Assessing integration across all relevant systems can help resolve incongruencies and proactively enact strategies that support technology transfer.

Several system-level factors have been reported in the literature that influence integration of technology-based interventions in disability or rehabilitation practice. Reviews identify a lack of intervention fit within organizational capacities and workflows as important barriers to technology adoption, which may include referral processes [100], scheduling [98,100], service delivery models [100], and length of therapy sessions [98,100]. Organizational culture and policy, particularly regarding technology in practice, is another important factor that can facilitate or hinder integration [99,100].

The Agency for Healthcare Research and Quality Implementation Feasibility Assessment evaluates integration factors by having service providers rate intervention fit with organizational capabilities (Criterion 1) and alignment with policies and incentives (Criterion 2) [106]. The Program Sustainability Assessment Tool can be used to assess organizational structures and processes that promote sustained integration [109]. From the perspective of clients and families, the ease of integrating a technology-based intervention in their home environment and daily routine is a priority [110].

### Impact

Performance expectancy, or perceived usefulness, is a leading determinant of acceptance and use of technology-based interventions among therapists, caregivers, and people with disabilities [101,111,112]. The impact dimension evaluates the promise or potential that a technology-based intervention shows in eliciting positive change for its target population. This dimension has been renamed from *limited efficacy* described by Bowen et al [12] to recognize the diverse aims of technology-based interventions and methods through which their benefits may be evaluated. Although the focus of feasibility research is process rather than outcomes, this stage is an opportunity to gather preliminary evidence on participant responses to the intervention [11,12,18,19].

Various research designs involving quantitative, qualitative, or mixed methods approaches may be used at the feasibility stage to explore potential impacts of technology-based interventions. Preliminary effects can be measured using descriptive observational studies, case reports or series, single-subject designs, interrupted time series designs, or one-group pretest-posttest designs [4]. Outcomes of interest for care recipients include symptoms, functional performance, participation, mobility, quality of life, and other psychosocial constructs [57]. The purpose of evaluating impact during the feasibility stage is not to definitively establish effectiveness but rather to test assessment procedures that accompany the intervention, evaluate appropriateness and sensitivity of outcome measures, and inform features of intervention design including duration and dosage [11]. Qualitative feedback is essential to accomplish these goals by exploring user perceptions regarding perceived benefits and the meaningfulness of outcome measures [75].

For care providers, the usefulness of technology in supporting care is an important driver of adoption [101], indicating that perceived job performance or quality of care are relevant measures of impact. For technologies designed to reduce therapist workload, such as autonomous robots and exoskeletons, impact may also be measured in subjective task effort or stress and burnout, which are common among care providers [113]. Alleviating caregiver burden is a target of some interventions for which impact may be investigated using the Caregiver Assistive Technology Outcome Measure [114]. System-level impacts are also relevant to technology-based interventions, which may include measures of resource use, service efficiency, and access to care [115-117].

### Ethicality

Ethical concerns are barriers to adoption and commercialization across a variety of technology-based approaches in disability and rehabilitation [118-120]. Common ethical concerns include challenges related to privacy, security, equity, stigma, autonomy, and power [118-120]. There are calls for more proactive consideration of ethics during development, evaluation, implementation, and policymaking processes for technology-based interventions [120]. The ethicality dimension considers the degree to which a technology-based intervention aligns with values of biomedical and social ethics. Ethical evaluation during feasibility studies can help anticipate and address concerns that may affect eventual adoption or policy [4].

Ethical concerns occur in the field of disability and rehabilitation when there is conflict between two or more biomedical or societal values. For example, protecting the safety of older adults using smart home monitoring technologies while maintaining personal privacy [121]. Applied ethics frameworks outline considerations for identifying and navigating these conflicts. Principles of bioethics and technology ethics may be useful for identifying value conflicts among technology-based interventions. Bioethics, concerned with ethical issues related to biomedicine and health care, includes 4 primary principles: (1) autonomy (personal choice), (2) beneficence (provision of benefit), (3) nonmaleficence (avoidance of harm), and (4) justice (fairness and equity) [122]. Technology ethics address issues related to technological advancements; relevant concepts overlap with bioethics and include human welfare, ownership and property, privacy, freedom from bias, universal usability, trust, autonomy, informed consent, accountability, identity, calmness, and environmental sustainability [123].

Based on these concepts, ethical domains relevant to the intervention can be compiled in consultation with users and other interest holders [121]. As the intervention is trialed during feasibility testing, conflicts across these domains can be monitored through discussion with users and reflective questioning about how the technology is used and any unexpected secondary effects [121]. A Socratic approach to questioning can be used, which poses morally relevant questions to highlight value issues related to health technologies [124]. The Model for Ethical Evaluation of Sociotechnical Arrangements (MEESTAR) is another tool to guide dialog on ethical analysis, designed specifically for assistive technologies [125]. It comprises seven ethical dimensions (care, autonomy, safety, justice, privacy, participation, and self-conception) across four levels (harmless, ethically sensitive with possibility of compensation, extreme ethical sensitivity requiring monitoring, opposition of use) and three perspectives (individual, organizational, society). Key questions are provided to support ethical discussion across the seven dimensions.

An example of ethical evaluation during feasibility testing can be found in a study from Kernebeck et al of an app- and sensor-based assistive technology intervention for caregivers of people with dementia [126]. In addition to exploratory outcome measures, the study involved a process evaluation of implementation, mechanisms of impact, and intervention context, including ethical implications. The ethical evaluation was a workshop based on MEESTAR conducted after the intervention with the study team, interest holders, and advisory board members. The authors highlight how this process supports the design of their intervention to reflect appropriate ethical orientation considering the values of independence, privacy, and safety from multiple perspectives [126].

### Commercial Viability

Cost and affordability are priorities in decisions regarding rehabilitation and assistive technology interventions from the perspective of clinicians, care recipients, families, health care organizations, and policymakers [98,99,127,128]. Yet, economic factors are infrequently and inadequately captured in research on these interventions [129]. The commercial dimension evaluates the degree to which a technology-based intervention shows promise toward being economically viable for commercialization. While often reserved for later implementation stages, gathering data on economic considerations at the feasibility stage can help refine intervention features to better fit the market, be more cost-efficient, and support commercialization.

Various economic evaluation methods are described in the complex intervention and health technology assessment literature. Cost-utility and cost-effectiveness analyses compare the costs and health outcomes of a new intervention against a comparator (eg, current standard of care), but use different measures of health outcome [130]. Cost-effectiveness analyses employ natural units of clinical effect, such as life-years or cases of disability, while cost-utility analyses, a subtype of cost-effectiveness analysis, use generic measures of health status reflecting both morbidity and mortality (eg, disability-adjusted life-years) [130]. Cost-benefit analyses express costs and benefits in financial terms and aim to capture a broader range of economic costs, including those not directly related to health outcomes (eg, cost of travel to care) [130]. Cost-utility analyses are favored in policy decision-making regarding health technologies because they allow comparison across conditions and intervention approaches [128].

The comprehensive economic evaluations described above may not be appropriate at the feasibility stage. During early development and testing of health technologies, Sculpher et al [131] recommend that economic evaluation should focus on defining and understanding the costs of existing practice that the new technology may help alleviate, and exploratory economic modeling of potential cost-effectiveness using data from existing studies or limited prospective data collection [131]. Relevant costs to consider for assistive and rehabilitation interventions include device production, assessment and training services, staffing, and servicing or maintenance. In terms of economic value, cost savings can be characterized through factors such as improved health outcomes, reduced costs of care, increased productivity, and decreased caregiver burden [129]. These exploratory economic feasibility approaches can inform intervention refinement by helping to redesign device features using more cost-efficient methods and improve efficiency of delivery services.

## Discussion

### Overview

Feasibility studies are of growing importance in intervention research and frameworks are available to guide this work across health disciplines, yet none specifically address technology-based interventions, which are inherently complex and necessitate iterative development and evaluation approaches [4]. This viewpoint helps define the scope of feasibility studies for technology-based interventions and highlights considerations for this work in the field of disability and rehabilitation. Key contributions and practical uses are discussed below.

### Contributions and Recommendations

Chiefly, this article is intended to support researchers and developers (in academic, clinical, or industry settings) by providing clarity on terminology in feasibility studies of technology-based interventions. Reviews of feasibility research across health disciplines have identified inconsistent use of terminology and variability in methods of evaluation [13,20]. Synthesizing concepts from feasibility literature and research on rehabilitation and assistive technology interventions, we outline various dimensions of feasibility specifically relevant to technology-based interventions and highlight potential criteria for their evaluation (Table 1). This can inform the design of comprehensive feasibility studies that reflect unique technological considerations and the complex factors that influence rehabilitation intervention outcomes. Integrating engineering practices and digital health concepts, we collated conceptual definitions and practical examples (Tables 2 and 3) to facilitate dialog between technical developers and clinical research teams. The compiled measurement instruments (Textboxes3  4) can help tailor data collection approaches and tools for specific types of technology-based interventions. Ultimately, our contributions serve to harmonize terminology and evaluation methods, enable critical selection of feasibility dimensions and criteria, and facilitate interpretation and comparison across studies.

To our knowledge, this is the first attempt to consolidate information on feasibility studies specifically for technology-based interventions. While further refinement may occur in time, this work provides essential background material to support study design decisions. Those seeking to apply information in this article should be aware of general best practices for feasibility studies [10,12,17]. We recommend that teams carefully select and justify the most appropriate dimensions and evaluation criteria according to their project needs, relevant uncertainties, and field-specific considerations (Textbox 2). These decisions are influenced by various contextual factors like intervention maturity, risk profile, and setting; Multimedia Appendix 1 provides sample reflection questions to support critical decision-making. Setting *a priori* success criteria for each feasibility dimension promotes rigor within and between studies for evidence building. Importantly, failure to achieve criteria may signal the need for intervention refinement (eg, software improvements to enhance usability). The process of feasibility evaluation is thus highly iterative, whereby multiple rounds of testing may be warranted to progressively refine intervention prototypes until comprehensive success criteria are met [4]. For instance, it may be appropriate to first establish user acceptability and demand with early-stage prototypes for interventions involving technology that is expensive or difficult to produce, while high-risk interventions with potential harms or discomforts necessitate early focus on safety and technical performance.

This work is also relevant for improving alignment between research and policy on technology-based interventions. The feasibility dimensions we delineate align with and extend current practices in health technology assessment—the comprehensive review process that informs policy recommendations on adoption of new technologies in health systems [127]. While health technology assessment approaches differ between jurisdictions, there is a common shared focus on considering factors beyond only clinical evidence on safety and effectiveness [127,132]. Health technology assessment processes around the world recognize the importance of organizational integration, economic and resource efficiency, social implications, equity, ethical ramifications, and user and public perspectives in decision-making [127,132]. These multidimensional factors and their application to technological interventions are not specifically articulated in existing feasibility literature and thus may not be considered until their implementation, after significant resources have already been invested in development and outcome evaluation. By considering these factors in the early stages of evaluation, potential issues can be proactively identified and addressed through refinement of intervention features and delivery approach, which we emphasize as a key aim of feasibility studies for technology-based intervention. In doing so, innovations can be advanced with the greatest likelihood of attaining policy support for widespread adoption to the benefit of people with disabilities or rehabilitation needs and the communities that support them.

### Future Directions

The ideas in this viewpoint represent the experiences and opinions of a small authorship team. While a useful guide for research and development, it is a starting point for future work. There is a need for broader discussion and engagement among interest holders in this space to establish consensus on feasibility considerations and dimensions in this field. It is our hope that this article serves as a catalyst for such dialog. Future directions building on this work may include the development of decision aids (eg, checklists, workflows, and case examples) and reporting guidelines to ensure adequate consideration, justification, and operationalization of relevant feasibility issues during the intervention development process. Decision tools may also help guide the process of iteratively refining and advancing intervention prototypes. Methods of weighting or prioritizing feasibility dimensions could also be useful in the context of limited resources or competing priorities.

## Supplementary material

10.2196/79026 Multimedia Appendix 1 Sample feasibility reflection questions.

## Abbreviations

EMA: Ecological momentary assessment
FASTER: Framework for Accelerated and Systematic Technology-based intervention development and Evaluation Research
ISO: International Organization for Standardization
MRC: Medical Research Council
RCT: randomized controlled trial
TAM: Technology Acceptance Model
tDCS: transcranial direct current stimulation
UTAUT: Unified Theory of Acceptance and Use of Technology
