# Supplementary material for: Evaluating the Feasibility of Technology-Based Interventions in Disability and Rehabilitation: Definitions, Considerations, and Dimensions
Source: JMIR Rehabil Assist Technol. 2026 Apr 14;13:e79026. doi: 10.2196/79026 (PMC13078705; doi:10.2196/79026)
Supplement: Multimedia Appendix 1 [file rehab-v13-e79026-s001.docx]

**Appendix 1.** Sample feasibility reflection questions to support critical decision-making.

| **Example influencing factors** | **Example reflection questions** |
| --- | --- |
| Intervention maturity | - What level of fidelity (e.g., low, medium, high) is our prototype, and which aspects may require further design refinement? - How confident are we that the technology will function as expected and satisfy its technical requirements in the target context(s)? - Which key support services should be evaluated alongside the intervention? |
| Users | - Do we have evidence that users judge the intervention favorably and are likely to use it as intended? - What intervention modifications are available to meet the diverse needs of target users and use contexts? - Have we demonstrated that users can successfully achieve their goals when using the intervention? - Have we established what meaningful intervention impacts would be for the target population(s)? |
| Setting | - Have we documented the resources required for intervention use and do they align with those available in the target setting(s)? - How confident are we that the intervention can be deployed and used as intended in its target setting(s)? - What existing systems will need to incorporate the intervention and how confident are we of the interventions “fit”? - Are there social, ethical, or security concerns associated with intervention use in the target setting(s) that should be explored? |
| Risk profile | - Are there potential harms or discomforts associated with the intervention that should be prioritized for early evaluation? - Is the intervention risk affected by any mitigating or aggravating factors associated with the users or context of use? - Should safety be established through initial testing with a lower-risk population? |
